# EphB2-mediated ephrin-B reverse signaling on microglia drives an anti-viral, but inflammatory and neurotoxic response associated with HIV

**DOI:** 10.1186/s12974-025-03481-9

**Published:** 2025-06-30

**Authors:** Jeffrey Koury, Hina Singh, Samantha N. Sutley-Koury, Dominic Fok, Xinru Qiu, Ricky Maung, Benjamin B. Gelman, Iryna M. Ethell, Marcus Kaul

**Affiliations:** 1 Division of Biomedical Sciences, School of Medicine, University of California, Riverside, 900 University Ave, Riverside, CA 92521 USA; 2 Infectious and Inflammatory Disease Center, Sanford Burnham Prebys Medical Discovery Institute, 10901 North Torrey Pines Road, La Jolla, CA 92037 USA; 3 Division of Genetics, Genomics and Bioinformatics, University of California, Riverside, 900 University Ave, Riverside, CA 92521 USA; 4 Department of Pathology, University of Texas Medical Branch, 301 University Blvd, Galveston, TX 77555-0419 USA; 5 Department of Neuroscience and Cell Biology, University of Texas Medical Branch, 301 University Blvd, Galveston, TX 77555-0419 USA

**Keywords:** Neuroinflammation, HIV-1, Ephrin-B, EphB, Interferon-β, Neurotoxicity, Microglia

## Abstract

**Background:**

Pathological inflammation with a loss of synaptic integrity and function has been implicated in HIV Associated Neurocognitive Disorders (HAND). Although therapeutics exist to increase the lifespan of people living with HIV (PLWH), they are not effective at preventing neuroinflammation and HIV induced neuronal damage persists. In this study, we investigate the ephrin-B/EphB axis, which regulates inflammation, in post-mortem brain specimen of PLWH and experimental models in order to assess its potential role in HIV induced neuroinflammation.

**Methods:**

We analyze mRNA samples of post-mortem brain specimen of PLWH and uninfected controls obtained from the National NeuroAIDS Tissue Consortium (NNTC) and, for comparison, of a transgenic mouse model of neuroHIV using quantitative reverse transcription polymerase chain reaction (qRT-PCR). Follow-up experiments employ mouse brain tissue and in vitro models, including immortalized human microglia, human induced pluripotent stem cell (iPSC)-derived mixed neuroglial cell cultures, cellular and molecular interference, functional and multiplex assays, immunofluorescence and mRNA sequencing to examine the role of the ephrin-B/EphB axis in neuroinflammation and the associated neurotoxicity.

**Results:**

Using qRT-PCR we find increased expression of EphB2 in post-mortem brain of PLWH, and detect a correlation with pro-viral DNA, viral RNA and an inverse correlation with abstract executive function and verbal fluency. Increased expression of ephrin-B/EphB at mRNA and protein level is also observed in brains of a transgenic mouse model of neuroHIV suggesting the upregulation can be driven, at least in part, by expression of viral gp120 envelope protein and a type I interferon, IFNβ. Additionally, we find induction of ephrin-B1 expression in microglia following activation by IFNβ. Given the previously reported impact of EphB2 on inflammation in the periphery, the functional role of EphB2-mediated ephrin-B reverse signaling on microglia is assessed for a pro-inflammatory and anti-viral signature. We find that EphB2 treated microglia secrete inflammatory and anti-viral factors but also exert contact-independent neurotoxicity. Finally, knockdown of microglial ephrin-B1, an EphB2 binding partner, shows a partial alleviation of the microglial pro-inflammatory signature and neurotoxicity.

**Conclusion:**

Our study suggests that elevated EphB2, and its reverse signaling through ephrin-B1 in microglia contribute to neuroinflammation and neurotoxicity in neuroHIV.

**Graphical Abstract:**

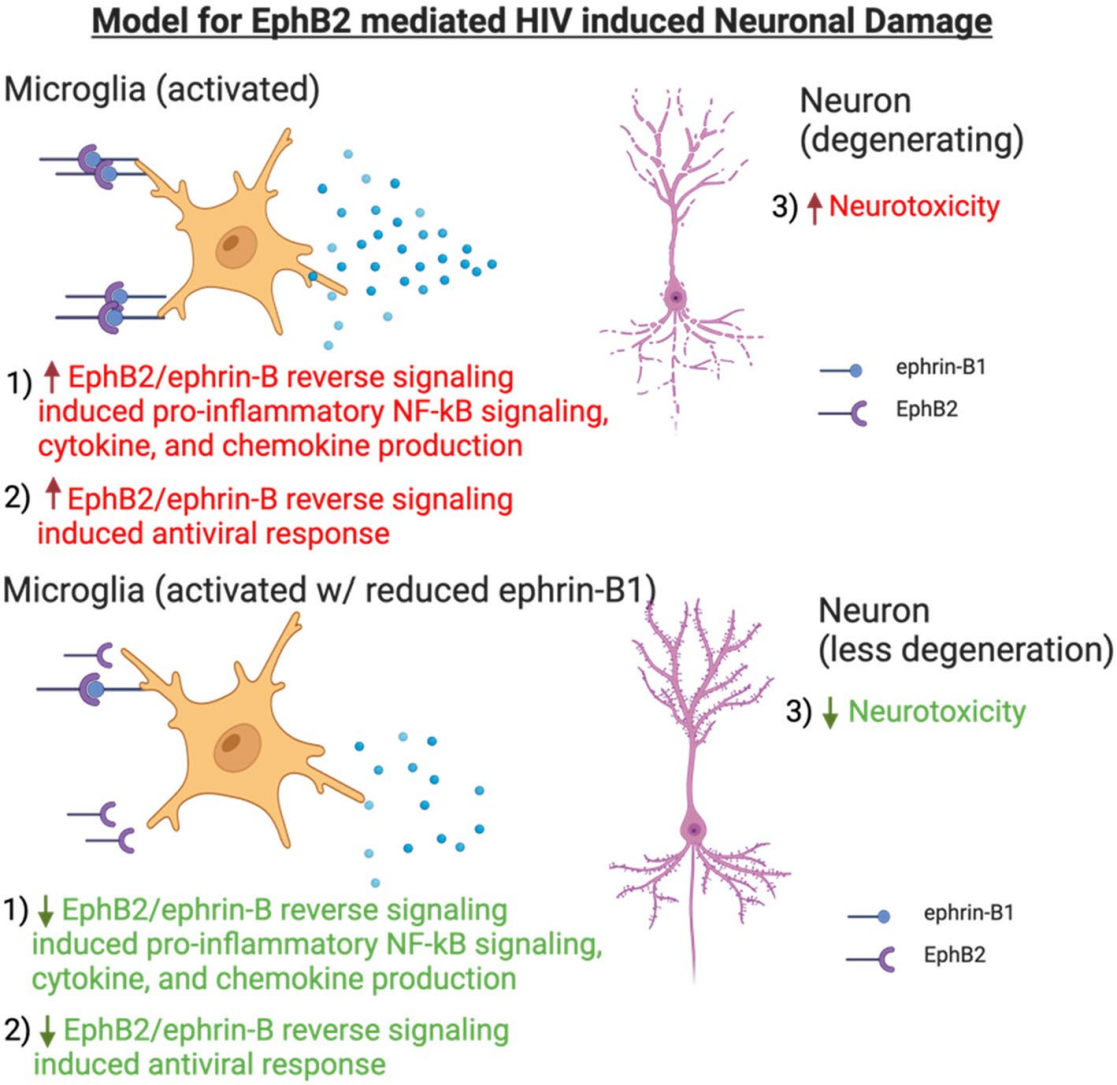

**Supplementary Information:**

The online version contains supplementary material available at 10.1186/s12974-025-03481-9.

## Background

HIV-associated neurocognitive disorder (HAND) is a condition characterized by cognitive and neurological impairments that occur in HIV infection and is found in up to 55% of people living with HIV (PLWH) [1]. Symptoms range from mild cognitive deficits to severe dementia, manifesting in various ways, including deficits in memory, attention, concentration, language, decision-making and motor skills, with or without concurrent depression [2]. The exact mechanisms behind HAND and HIV induced neuronal injury are not fully understood, but several factors presumably contribute to its development, namely chronic inflammation, release of viral proteins and glutamate excitotoxicity [3]. Additionally, it is widely accepted that macrophages and microglia are key mediators of HIV induced inflammation and neurotoxicity. Notably, one pathological characteristic of neuroHIV is the presence of multinucleated microglial cells [4]. Furthermore, depletion of microglia alone is sufficient to abrogate HIVgp120 induced neurotoxicity in a HIV mouse model and primary cerebrocortical cultures [5–7]. Additionally, leveraging a transgenic mouse model expressing HIV glycoprotein gp120 (HIVgp120tg) [8], in combination with analysis of human tissues, our previous studies have implicated several components of the innate immune system, specifically the type I interferon response, in neuroHIV [7, 9–11]. Here we identify a novel mechanism driving neuroinflammation and toxicity mediated by activation of the ephrin-B/EphB axis that is associated with anti-viral responses in neuroHIV.

Ephrins and Eph receptors belong to a family of receptor tyrosine kinases that play crucial roles in various physiological processes, including embryonic development, neural connectivity and, although less recognized, inflammation [12]. Both ephrins and Eph receptors are membrane-bound proteins, which interact in a cell–cell contact dependent manner and have a unique ability to signal bidirectionally. As it pertains to inflammation in the CNS, EphB2 deletion in a murine stroke model was reported to decrease proinflammatory CCL2 and IL-6 in the ischemic zone, while EphB2 binding to astrocytic ephrin-Bs activated NF-kB signaling, suggesting a chronic inflammatory role for EphB2 [13]. The role of astrocytic ephrin-B1 was also reported in a mouse model of traumatic brain injury (TBI), showing that astrocyte-specific ablation of ephrin-B1 suppressed TBI-induced STAT3 phosphorylation while the activation of ephrin-B1 in astrocytes induced upregulation of STAT3 [14]. However, the functional role of EphB/ephrin-B signaling in microglia has not been explored although it could be of particular importance in the context of neuroinflammation and neurodegenerative disease as microglia are key inflammatory mediators in the CNS [15, 16].

In this study, we highlight the elevated expression of EphB2 in both the CNS of PLWH and in HIVgp120tg mice and report an up-regulation of ephrin-B1, specifically on microglia, in the hippocampus and cortex of HIVgp120tg animals. Our data support the pro-inflammatory impact of EphB2 reverse signaling and the role of microglial specific ephrin-B1 in mediating an anti-viral but also inflammatory response. Finally, we show that EphB2 mediated ephrin-B activation in microglia triggers the release of neurotoxins affecting neuronal survival in iPSC derived neuronal/astrocytic co-cultures. Altogether, our study suggests that elevated EphB2 levels in the CNS of PLWH contribute to the chronic inflammation, driving at least some of the neuropathology associated with HAND, and that therapeutics targeting EphB2-mediated ephrin-B signaling in microglia may minimize these deleterious effects [17].

## Results

### EphB2 is differentially expressed in PLWH with brain pathology and its levels correlate with impaired cognitive performance

**Fig. 1:**
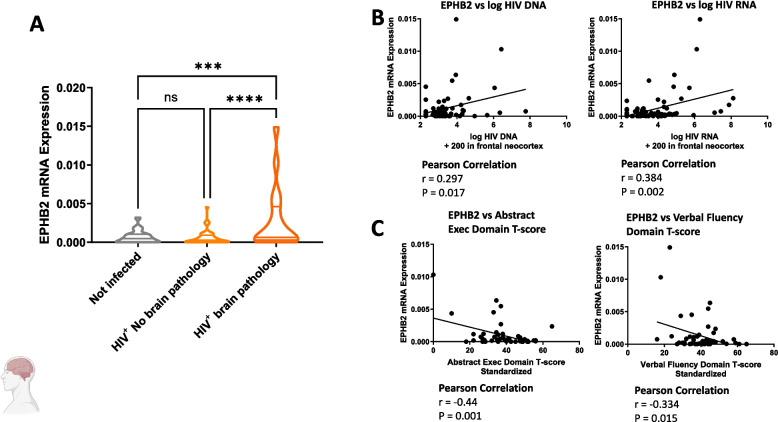
EphB2 is differentially expressed in human neocortex of PLWH with brain pathology. **A** Relative gene expression levels of EphB2 using mRNA from the middle frontal gyrus (neocortex) of the following groups of individuals: Not-infected (*n* = 46) and HIV + (*n* = 63), including HIV^+^ no brain pathology (*n* = 44) and HIV^+^ brain pathology (*n* = 19). The relative gene expression levels of EPHB2 in HIV^+^ (*n* = 63) patient’s cortex was used to calculate Pearson correlations for (**B**) log HIV DNA/RNA load (+ 200 represents the threshold of HIV DNA/RNA detection in the assay) and (**C**) two domains of neurocognitive performance**.** Values in graph are mean ± SEM; n.s., non-significant, * *P* ≤ 0.05, ** *P* ≤ 0.01, *** *P* ≤ 0.001, **** *P* ≤ 0.0001, One-Way ANOVA followed by Tukey’s post hoc test. Schematic graphics created with Biorender software

Given that neuroinflammation is a hallmark characteristic of neuroHIV, and previous studies of other diseases implicate EphB2 in inflammation [18, 19], we first investigated, if EphB2 is elevated in the CNS of PLWH by analyzing mRNA expression in neocortex (middle frontal gyrus) of a previously characterized cohort [20]. The specimen from this cohort comprised PLWH (*n* = 63), including PLWH without brain pathology (*n* = 44) and PLWH with brain pathology (*n* = 19), and non-infected controls (*n* = 46). The analysis of EphB2 mRNA expression shows that its levels are preferentially elevated in PLWH with brain pathology (*p* < 0.0001) compared to controls and PLWH without brain pathology (Fig. 1A). In this context, brain pathology is defined by the presence of multinucleated giant cells or microglial nodules, highlighting microglia as a potential key target and/or cellular source for EphB2. In PLWH, EphB2 mRNA levels positively correlate with log HIV DNA (*r* = 0.297, *p* = 0.017) and log HIV RNA load (*r* = 0.384, *p* = 0.002) (Fig. 1B). Additionally, levels of EphB2 mRNA inversely correlate with performance in pre-mortem neurocognitive tests, described by the abstract executive domain T score (*r* = −0.44, *p* = 0.001) and verbal fluency domain T score (*r* = −0.334, *p* = 0.015) (Fig. 1C). These findings support a role for EphB2/ephrin-B signaling in brain pathology, increased viral load, and poorer cognitive scores in PLWH. As brain pathology is characterized by a pathological presentation of microglial nodules or multinucleated giant cells, we hypothesized that EphB2 may be acting specifically on microglia.

### IFNβ and IRF7 regulate ephrin-B/EphB in cortex and hippocampus, and microglial cells

**Fig. 2:**
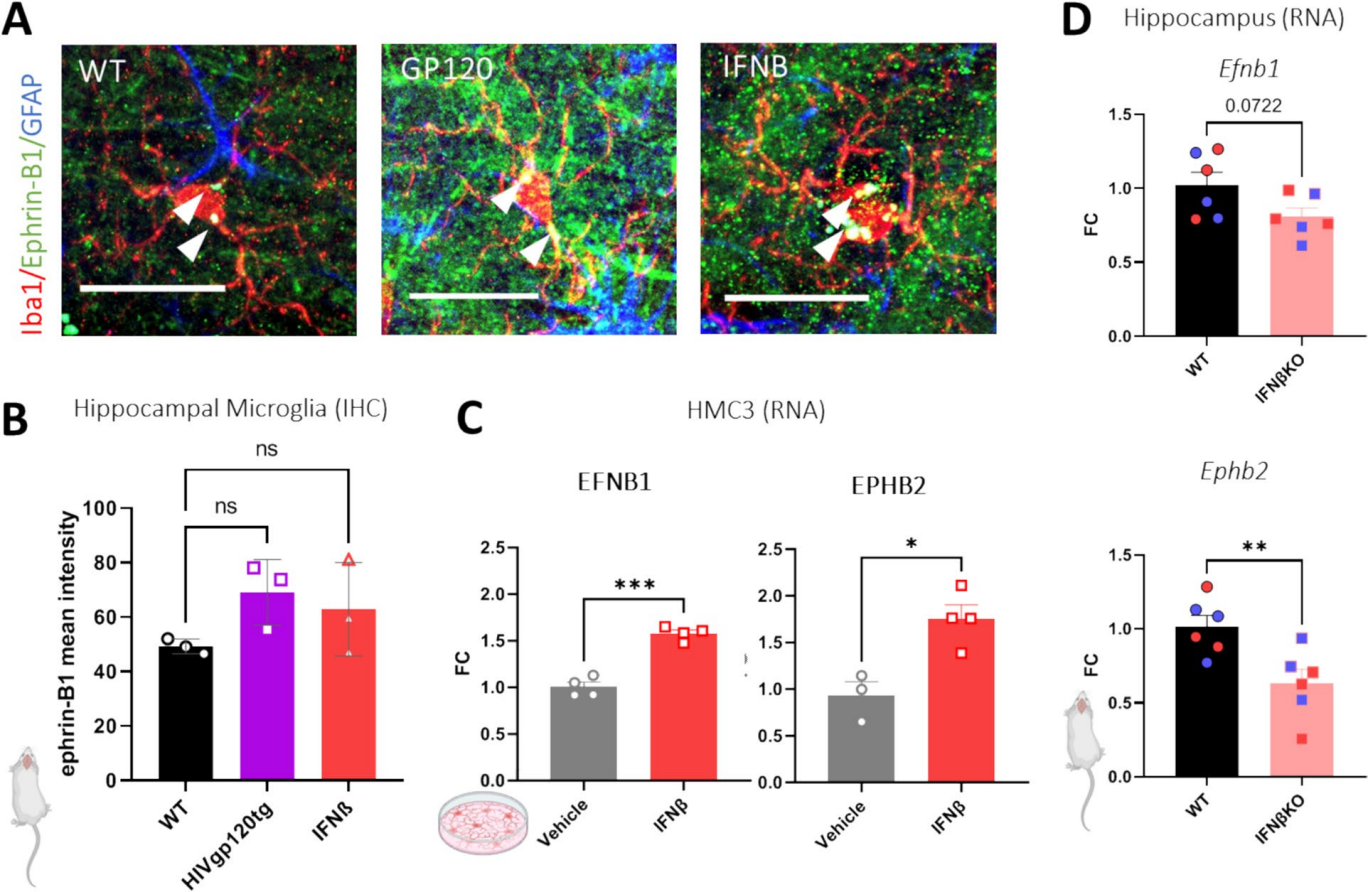
HIVgp120 and IFNβ regulate total and microglial specific ephrin-B1 expression in hippocampus. **A** Sagittal brain section stained for astrocytic GFAP (blue), microglial Iba1 (Red) and ephrin-B1 (Green). Scale bar = 20um. **B** Values are Mean Intensity of ephrin-B1 fluorescence in CA1 hippocampal microglia. All mice for IHC were male, aged 3–4 months. IFNβ indicates intranasal treatment with the cytokine. Each data point represents *n* = 3 animals per genotype, with an average of 3 sections per animal and 2 images per section. Values are Mean + SD; FC = Fold Change; n.s. = non-significant (*P* > 0.05), Two Way ANOVA followed by Tukey’s post hoc test. **C** Increase in EFNB1 and EPHB2 mRNA expression following 24-h IFNβ treatment in human microglial HMC3 cells; *n* = 3–4 experiments, Two technical replicates averaged per experiment. Values are Mean ± SEM; FC = Fold Change; n.s. = non-significant, * *P* < 0.05, ** *P* < 0.01, *** *P* < 0.001, **** *P* < 0.0001, students t-test. **D** Analysis of mRNA expression of ephrin genes in the hippocampus of WT and IFNβKO. Relative mRNA expression was calculated by normalizing to *Gapdh* using the 2^−ΔΔCt^ method. RNA of IFNβKO and WT mice for qRT-PCR was from hippocampus of males and females, *n* = 6 (male *n* = 3 – blue data points; female *n* = 3 – red data points) per genotype. Values are mean ± SEM; n.s., non-significant, * *P* ≤ 0.05, ** *P* ≤ 0.01, *** *P* ≤ 0.001, **** *P* ≤ 0.0001, One-Way ANOVA followed by Tukey’s post hoc test. Schematic graphics created with Biorender software

EphB2 and ephrins signal bidirectionally and we utilized the HIVgp120tg mouse model of neuroHIV to assess the cell specific expression of the binding partner of EphB2, ephrin-B1, which can act as both a ligand and a receptor. In conjunction, we sought a potential mechanism that may be regulating ephrin-B/EphB expression in the CNS. To investigate whether HIV, or viral protein expression, which trigger a type I interferon response, or the interferon response itself can regulate ephrin-B/EphB expression, we assessed the mean intensity of ephrin-B1 immunoreactivity in the CA1 hippocampus. We specifically examined in Iba1^+^ microglia, in 3–4 month-old mice, constitutively expressing the HIV envelope protein gp120 or treated intranasally with recombinant IFNβ (a type I interferon) as recently published [9]. HIVgp120tg animals show a prominent type I interferon response, including activation of interferon regulatory factors (IRFs) and anti-viral interferon stimulated genes (ISGs). Transgenic HIVgp120 expression elicits a type I interferon response, and so both HIVgp120tg mice and IFNβ treated non-tg mice were assessed to determine the potential role of the type I interferon response in the regulation of ephrin-B1/EphB levels. Indeed, both the HIVgp120 transgene and IFNβ treatment alone showed an increased average level in the expression of ephrin-B1 in microglia of the CA1 of the hippocampus, suggesting that the viral envelope protein gp120, and specifically the anti-viral response associated with its expression, may promote ephrin-B1 expression (Fig. 2A, B). While the differences observed in the mouse model did not reach significance, similar and significant effects were observed in human microglia. Treatment of HMC3 cells in vitro with recombinant human IFNβ (3,000 U/ml) induced elevated transcript levels of both ephrin-B1 (*p* < 0.001) and EphB2 (*p* < 0.05) approximately 1.5-fold compared to vehicle control (Fig. 2C). Inversely, ablation of IFNβ in the HIVgp120tg mouse model resulted in a significant decrease in both ephrin-B1 and EphB2 transcript levels in the hippocampus (Fig. 2D).

**Fig. 3:**
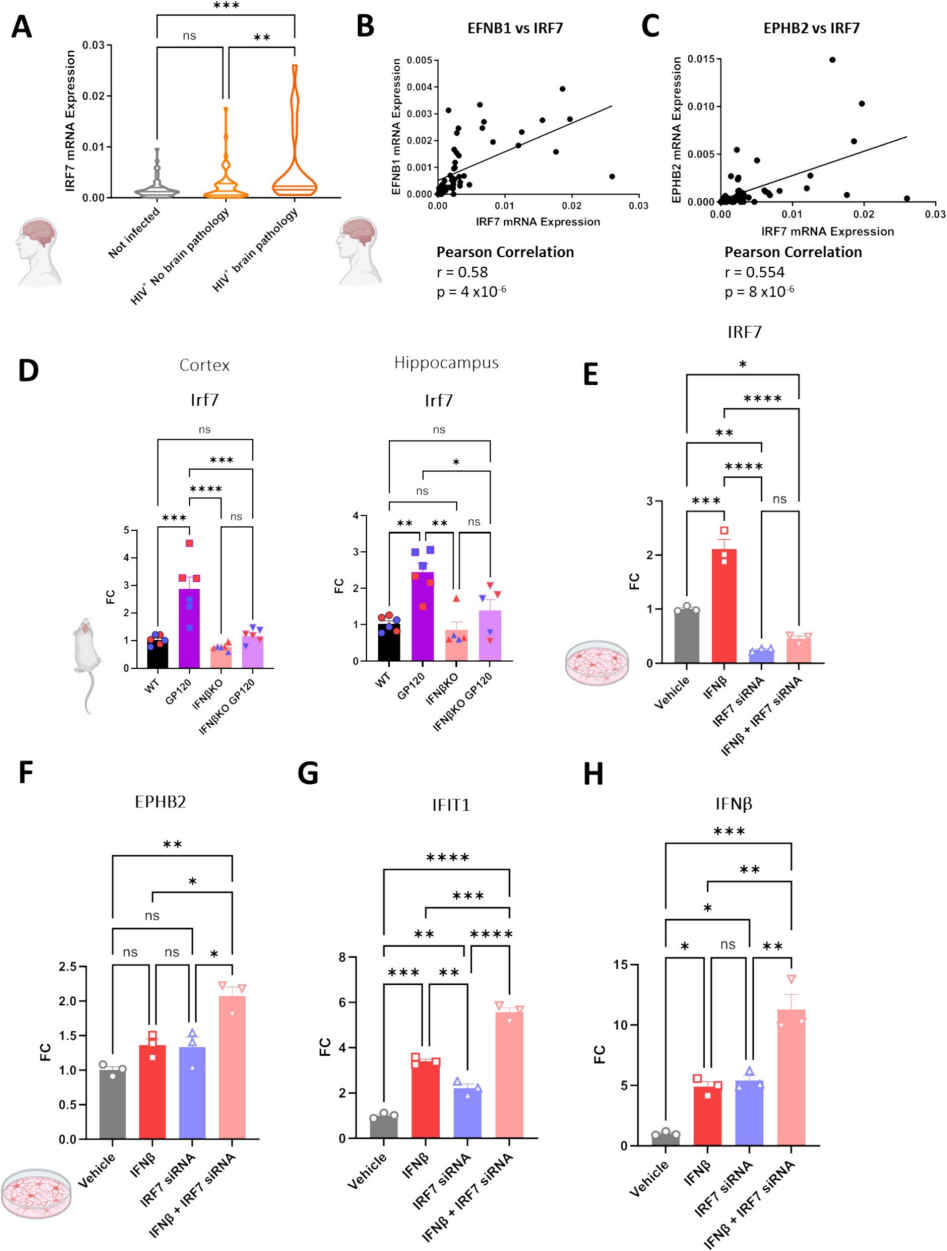
IRF7 regulates expression of type I interferon IFNβ, ISG IFIT1 and EPHB2. **A** Relative gene expression levels of IRF7 using mRNA from the middle frontal gyrus (neocortex) of Non-infected (*n* = 46) and HIV^+^ (*n* = 63), HIV^+^ No Brain Pathology (*n* = 44) and HIV^+^ Brain Pathology (*n* = 19). Relative gene expression levels of HIV^+^ (*n* = 63) patient’s cortex was used and Pearson correlations were calculated for IRF7 vs (**B**) EFNB1 (**C**) and EPHB2. (**D**) Relative gene expression levels of Irf7 from the cortex and hippocampus of 9-month-old WT, HIVgp120tg, IFNβKO and IFNβKO HIVgp120tg mice. *n* = 6 (*n* = 3 male – blue data points, *n* = 3 female – red data points) per genotype. Expression normalized to Beta-Actin. (**E**) IRF7, (**F**) EPHB2 (**G**) IFIT1 and (**H**) IFNβ mRNA expression following 48-h IRF7 siRNA or negative control siRNA treatment followed by 24-h IFNβ treatment or 0.001% BSA vehicle in human HMC3 cells; *n* = 3 biological replicates, 2 technical replicates averaged per experiment. Values are Mean ± SEM; FC = Fold Change; n.s. = non-significant, * *P* < 0.05, ** *P* < 0.01, *** *P* < 0.001, **** *P* < 0.0001, Two Way ANOVA followed by Tukey’s post hoc test. Schematic graphics created with Biorender software

Type I IFNs trigger the expression of interferon-stimulated genes, and we observed an increase in expression of IRF7 mRNA transcript levels in PLWH with brain pathology compared to uninfected controls, similar to the increase in EphB2 mRNA expression in the same group (Fig. 3A, *P* < 0.001 PLWH with brain pathology vs uninfected). Correlations in the neocortex of PLWH also revealed a very robust positive correlation between ephrin-B1 (Fig. 3B, *r* = 0.58, *p* = 4 × 10^–6^), EphB2 (Fig. 3C, *r* = 0.554, *p* = 8 × 10^–6^) and the type I interferon master regulator, IRF7, suggesting a strong association between ephrin-B/EphB and the type I interferon response. IRF7 transcript levels are also prominently induced in the cortex and hippocampus of the HIVgp120tg animals, and this upregulation is dependent on the activation of IFNβ signaling (Fig. 3D). With the discovery that IFNβ induced ephrin-B/EphB expression, and the strong correlation between the levels of ephrin-B1/EphB2 and the master regulator IRF7, we wanted to determine next if IRF7 was a key mediator in this IFNβ induced activation of ephrin-B/EphB. HMC3 human microglia were transfected with IRF7 siRNA (12.5 pmol) using RNAiMAX transfection reagent 48 h prior to treatment with IFNβ (3,000 U/mL) for 24 h. To our surprise, knocking down IRF7 in microglia (Fig. 3E) did not reduce expression of EphB2, but rather increase its expression, as well as that of other type I interferon genes, including IFIT1 and endogenous IFNβ itself (Fig. 3F-H). Moreover, knockdown of IRF7 also upregulated IRF3, the long-noncoding RNA HEAL and the transcription factor nuclear factor κB (NFκB) (Supplementary Fig. 1). Altogether, our observations suggested that IRF7 exerts a negative feedback on IFNβ and EphB2 expression and other factors involved in inflammation and regulation of HIV.

The data also suggests that the presence of HIVgp120 viral envelope protein and the type I interferon response, specifically IFNβ, is sufficient to induce ephrin-B/EphB expression in the CNS, including microglial cells. Although the functional role of EphB/ephrin-B signaling in neurodevelopment and the formation of synapses have been characterized, its role in CNS inflammation is poorly understood and previous studies describing the role of EphB2/ephrin-B have largely focused on the peripheral immune response [18, 21]. Thus, we assessed next to what extent elevated EphB2 in the CNS may be involved in inflammation and more specifically, if microglia are a cellular source of the resulting inflammation.

### EphB2-mediated ephrin-B reverse signaling induces a robust transcriptional anti-viral and pro-inflammatory signature in microglia

**Fig. 4:**
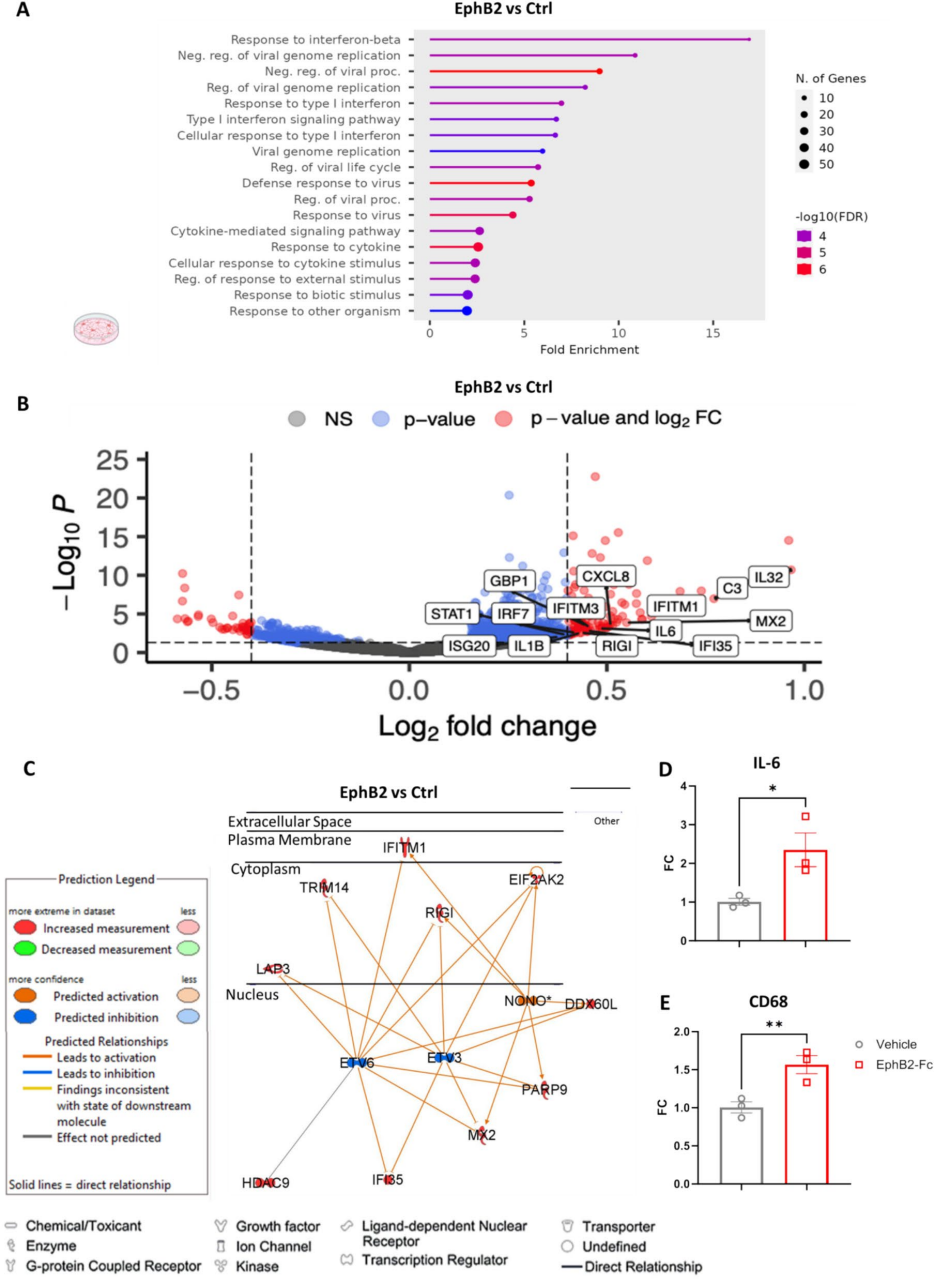
EphB2 reverse signaling in microglia activates anti-viral and inflammatory genes and pathways. Bulk RNA sequencing of HMC3 microglia treated with EphB2-Fc or Ctrl-Fc for 24 h. **A** GO Enrichment of EphB2 vs Ctrl using the top 300 upregulated genes. **B** Volcano plot of differentially expressed genes of EphB2 vs Ctrl samples (0.4 log2Fc and 0.05 p-value cutoff). **C** Network analysis using Ingenuity Pathway Analysis (IPA) of the second highest scoring network (anti-viral network). Blue indicates predicted down-regulated while red reflects up-regulated genes respectively. **D**-**E** mRNA transcript levels of IL-6 and CD68 from EphB2 treated microglia. RNA sequencing; *n* = 3 biological replicates. qRT-PCR; *n* = 3 biological replicates, 2 technical replicates averaged per biological replicate. Values are Mean ± SEM; FC = Fold Change; * *P* ≤ 0.05, ** *P* ≤ 0.01, *** *P* ≤ 0.001, **** *P* ≤ 0.0001, n.s., non-significant, student’s t-test. Schematic graphics created with Biorender software

To determine the function of EphB2/ephrin-B signaling in microglia, we treated human HMC3 cells with pre-clustered human recombinant EphB2-Fc (2ug/ml) for 24 h to induce ephrin-B reverse signaling. Subsequent RNA-sequencing (RNA-seq) revealed that human microglia treated with pre-clustered EphB2 exhibited a pro-inflammatory, anti-viral gene expression signature. GO enrichment analysis revealed upregulation of gene expression associated with biological processes, such as type I interferon response and cytokine mediated signaling and response (Fig. 4A). Assessment of highly differentially expressed genes (log2FC cutoff = 0.4, P value < 0.05) revealed a pro-inflammatory and anti-viral gene expression signature in EphB2- vs vehicle-treated microglia, including upregulation of IL-6, IFITM3, RIG-I, IRF7, and C3 (Fig. 4B, C). Network analysis showed a prominent activation of the type I interferon response and subsequent anti-viral response in parallel to the pro-inflammatory cytokine/chemokine response in EphB2-treated microglia. Moreover, transcript levels of specific inflammatory and myeloid activation genes were confirmed using qRT-PCR, including IL-6 and CD68, suggesting a pro-inflammatory/pro-phagocytic state for the microglia (Fig. 4D, E). Additionally, an about sevenfold increase of NF-kB phosphorylation was observed in EphB2- vs vehicle-treated microglia (that was not significantly reduced when ephrin-B1 was knocked down with siRNA treatment Supplementary Fig. 2 and 3), with canonical NF-kB related genes differentially expressed (i.e. IL-6, TNFα, IL-1B). Our findings highlight a novel function of ephrin-B reverse signaling, at least in microglia, namely, to prime the innate immune system by activating viral detectors (RIG-I), anti-viral effector proteins (IFITM3), complement (C3) and a master regulator of the type I interferon response (IRF7).

### EphB2-mediated ephrin-B1 reverse signaling in human microglia induces a pro-inflammatory secretome profile, and microglial ephrin-B1 knockdown mutes the response

**Fig. 5:**
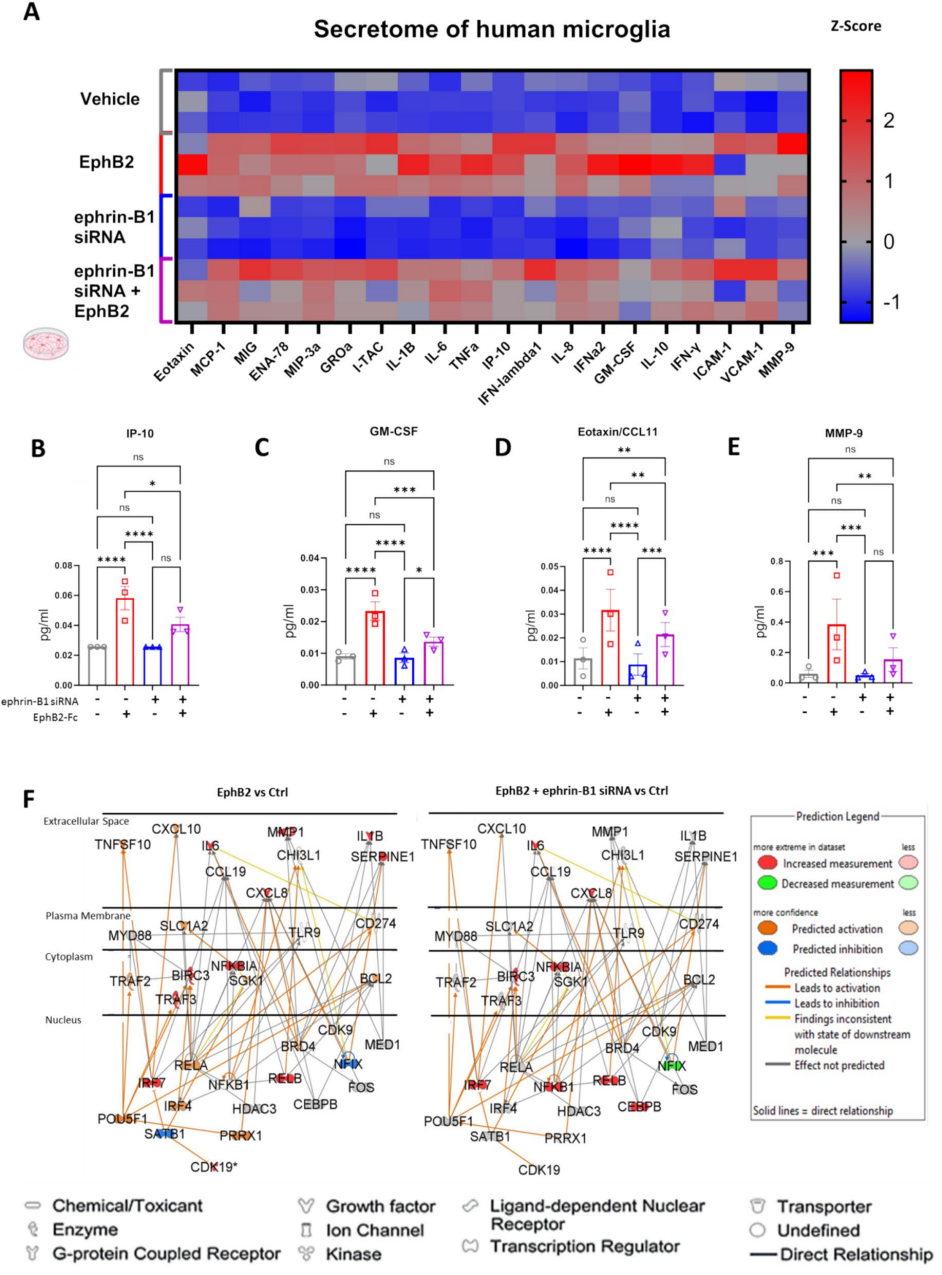
EphB2 reverse signaling induces a pro-inflammatory secretome profile, mediated in part by microglial ephrin-B1. **A** Multiplexed analysis of supernatants of human microglial HMC3 cells using LegendPlex bead assays following 48-h treatment with ephrin-B1 siRNA or scramble siRNA + 24-h EphB2-Fc (2ug/ml) or control-Fc (2ug/ml) exposure. *n* = 3 biological replicates. 2 technical replicates per biological replicate averaged. Heatmap values are z score based on averaged MFI of each technical multiplex replicate per experiment. **B**-**E** representative protein concentrations of IP-10, GM-CSF, CXCL1 and MMP-9 from the LegendPlex bead assay. **F** Network analysis for EphB2 vs Ctrl, and EphB2 + ephrin-B1 siRNA vs Ctrl (Ingenuity Pathway Analysis) to determine the network level changes from reducing ephrin-B1 during EphB2 mediated activation of microglia. Green indicates downregulated while red reflects upregulated genes, respectively. Blue indicates predicted down-regulated while orange reflects predicted up-regulated genes, respectively. Components without color represent genes without experimentally determined expression levels. * *P* < 0.05, ** *P* < 0.01, *** *P* < 0.001, **** *P* < 0.0001, n.s. = non-significant, Two-Way ANOVA followed by Tukey’s post hoc test. Schematic graphics created with Biorender software

The RNA-seq data revealed that EphB2 treatment of human microglia activated a multitude of genes downstream of the NF-kB signaling pathway, which is well recognized for its role as a transcriptional activator of pro-inflammatory cytokines [22]. Therefore, we examined the EphB2 induced profile of secreted products. Considering a significant increase in microglial ephrin-B1 immunoreactivity in the presence of HIVgp120 or IFNβ, we also assessed whether the effects of EphB2 on NF-kB signaling and cytokine responses in microglia are mediated through ephrin-B1 by knocking down microglial ephrin-B1. Ephrin-B1 was knocked down in human microglia via siRNA treatment for 48-h followed by a 24-h treatment of microglia with pre-clustered EphB2-Fc for secretome analysis (Fig. 5; Supplementary Fig. 2). Using the LegendPlex multiplex bead-based system, secreted cytokine and chemokine panels were interrogated at scale following EphB2-Fc and ephrin-B1 siRNA treatment of microglia. The results indicated that EphB2 mediated ephrin-B reverse signaling onto microglia drives an inflammation-related secretome profile (Fig. 5A), including IP-10/CXCL10 (Fig. 5B), GM-CSF (Fig. 5C), CCL11 (Fig. 5D), and MMP-9 (Fig. 5E) that is partially suppressed by microglial ephrin-B1 knockdown. In line with that finding, the second highest scoring gene network identified by Ingenuity Pathway Analysis (IPA) of microglial RNA-seq data shows, that compared to EphB2 stimulation alone, the reduction of microglial ephrin-B1 in the presence of EphB2 blunts activation of pro-inflammatory and NF-kB regulated molecules, including, IL1B, CXCL10, and RELA (Fig. 5F). Taken together, EphB2-mediated ephrin-B reverse signaling onto microglia results in secretion of pro-inflammatory cytokines and chemokines and this effect is partially prevented by the knockdown of microglial ephrin-B1, which significantly diminished secretion of IP-10 (CXCL10), GM-CSF, MMP-9 and CCL11 and caused a downward trend for IL-1β, IL-6, TNFα and CXCL1, −5, −11 (Supplementary Fig. 4). The only factor increased by the knockdown of ephrin-B1 was VCAM-1.

### Factors secreted from microglia following the treatment with EphB2 are sufficient to induce neurotoxicity

**Fig. 6:**
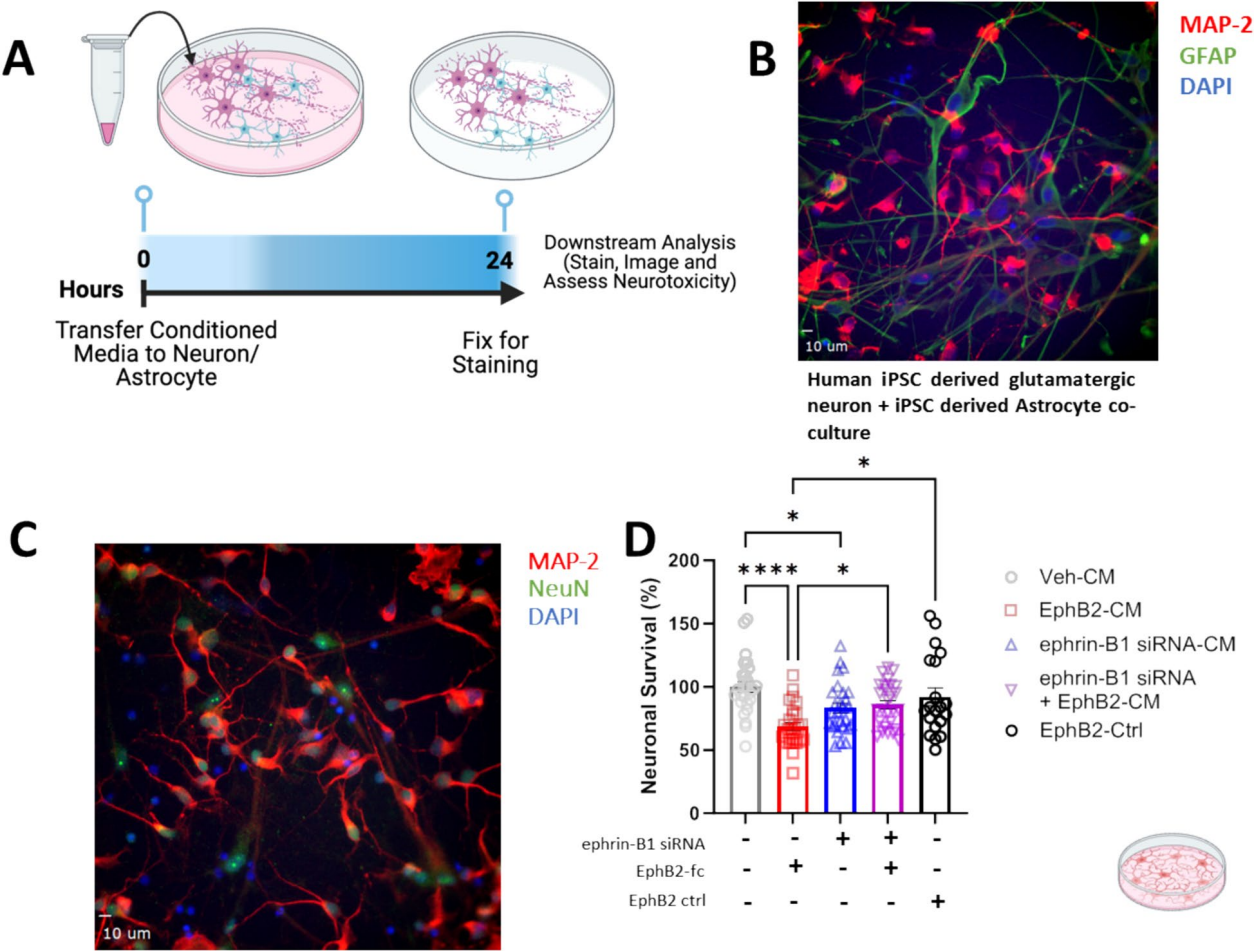
Secreted factors from EphB2 activated microglia induce neurotoxicity. **A** Timeline beginning with microglial conditioned media (CM) transfer. HMC3 microglia were previously treated for 48 h with RNAiMAX reagents and negative control (scrambled siRNA) or ephrin-B1 siRNA, then stimulated for 24 h with pre-clustered EphB2-Fc or ctrl-Fc before supernatants were collected. **B** representative image of Vehicle-CM treated iGluta/iAstrocyte (6:1 neuron to astrocyte) co-cultures stained with MAP-2 for dendrites (red), GFAP for Astrocytes (green) and Hoechst Dye (Blue). **C** The iGluta/iAstrocyte co-cultures were exposed for 24 h to 50% cell-free CM from treated microglia (image of Vehicle-CM treated cells). Neurotoxicity was assessed with MAP-2/NeuN double positive neuron counts following 24 h exposure to 50% cell-free microglia CM. **D** Co-cultures of iGluta/iAstrocyte exposed to media conditioned by vehicle-treated microglia (Veh-CM) were used to define 100% neuronal survival. Values are Mean ± SEM; *n* = 3 independent co-culture experiments, with CM from *n* = 3 independent HMC3 EphB2 stimulation experiments. Total neuron cell counts per treatment: Veh-CM = 1123, EphB2-CM = 765, ephrin-B1 siRNA-CM = 954, ephrin-B1 siRNA + EphB2-CM = 969, EphB2-Ctrl = 822. * *P* < 0.05, ** *P* < 0.01, *** *P* < 0.001, **** *P* < 0.0001, n.s. = non-significant, Two Way ANOVA followed by Tukey’s post hoc test. Schematic graphics created with Biorender software

Because the microglial secretome displayed a pro-inflammatory phenotype following EphB2 treatment and that effect was limited by microglial ephrin-B1 knockdown, we next assessed the impact of the respective microglial supernatants on neurons. Human iPSC derived glutamatergic neurons and human iPSC derived astrocytes were co-cultured (iGluta/iAstrocyte) at ratio of 6:1 as described in the method section (Fig. 6) and then incubated with 50% cell free conditioned media (CM) from vehicle-, EphB2-, ephrin-B1 siRNA- and ephrin-B1- siRNA + EphB2- and control siRNA-treated microglia. After 24 h, neurons were fixed, co-stained for MAP-2 (red) and NeuN (green), and neuronal survival was determined based on the counts of MAP-2/NeuN double positive cells (Fig. 6C, D). Neuronal survival was significantly reduced following incubation with EphB2 treated microglial CM. However, ephrin-B1 knockdown in microglia largely abrogated neurotoxicity induced by EphB2 treatment (ephrin-B1 siRNA + EphB2-CM vs EphB2-CM, *p* < 0.05), indicating that secreted microglial factors, such as pro-inflammatory components, stimulated by EphB2-ephrin-B1 signaling exert neurotoxicity.

## Discussion

Here we show first that components of the ephrin-B/EphB axis, namely EphB2 expression is elevated in the CNS of PLWH who display signs of brain pathology, but not those without brain pathology, highlighting EphB2 as a potential biomarker to discern the two groups. Second, components of the ephrin-B/EphB axis are on average elevated in the brain in association with expression of HIV viral RNA but also by treatment with exogenous recombinant IFNβ in a transgenic mouse model of HV brain injury. Third, in this model the EphB2 ligand/receptor ephrin-B1 is upregulated in microglia in the presence of HIVgp120 and after treatment with recombinant IFNβ (although the effect did not reach significance in the immunofluorescence-based analysis). Fourth, our study shows that EphB2-mediated ephrin-B reverse signaling induces, in microglia, activation of NF-kB and a pro-inflammatory and possibly anti-viral response in conjunction with neurotoxicity.

Previously, it was poorly understood what the regulators of ephrin-B/EphB in the CNS were, particularly in microglia, and the role of EphB2/ephrin-B signaling in microglia. Here, for the first time, we highlight HIVgp120 and IFNβ as potential key regulators of the expression of ephrin-B1, and inducers of EphB2. Expression of the type I interferon master regulator, IRF7, but not IRF1 or −3, correlates with ephrin-B1 and EphB2 in the cortex of PLWH and HIVgp120-transgenic mice, and is regulated by IFNβ in the mouse model. However, ablating IRF7 from human microglia and stimulating with IFNβ paradoxically resulted in enhanced activation of EphB2 and other anti-viral factors like IFIT1 and IFNβ transcripts. IRF7, among three other IRFs, IRF1, −3, and −5, operates as a regulator of the type I Interferon gene transcription [23]. However, it is possible the ablation of IRF7 resulted in the induction and activation of other IRFs, such as shown in our study for IRF3 transcript levels which may promote the activation of downstream ISGs and potentially EphB2. While we haven’t studied transcriptional regulation in details, it has been reported by others that the ratio of IRF3 to IRF7 impacts preference towards homo vs hetero-dimerization of the transcription factors, ultimately adjusting the landscape and binding affinity to interferon-stimulated response elements (ISREs) on downstream targets [24]. Ablation of IRF7 skews the ratio to IRF3, and thus presumably alters the landscape of activation leading to the potentiation of interferon-stimulated genes seen here, such as IFIT1, IFNβ itself and even EphB2. In any case, our observation suggests that IRF7 acts as a negative feedback on IFNβ and EphB2. Although IRF7 may not be mediating the IFNβ induced expression of EphB2, the ablation of IRF7 alone does induce transcription of IRF3, the long-non-coding RNA HEAL and NF-kB, three factors implicated in viral infection and inflammation [25, 26]. Moreover, one study has shown that EphB2’s regulatory region contains multiple binding sites for NF-kB near it’s transcriptional start site [19].

NeuroHIV, and the prevalence of HAND, can persist even when the viral load has become undetectable [2]. Anti-retroviral therapies extend the lifespan of PLWH and suppress viral replication; however, they do not eliminate the virus, thus allowing for viral protein products to continue to breakthrough and cause inflammation and neurodegeneration [2]. In a cohort of almost 2,000 PLWH, worse executive function was associated with lack of full virologic suppression [27]. The elevated EphB2 observed in the brains of PLWH who have brain pathology and the fact that EphB2 correlated with HIV DNA and RNA titers indicates a link with incomplete or failing viral suppression and may provide insights in the persistent neurocognitive problems. EphB2s inverse correlations with Abstract Executive and Verbal Fluency T-scores suggest a deleterious association of elevated EphB2 and neuropsychological performance, resembling conditions seen in non-virologically suppressed individuals and observed in another study [28]. This study reported that EphB2 expression was higher in neurocognitively impaired PLWH but did not distinguish cases with and without brain pathology or specify whether the individuals received antiretroviral therapy. Specifically, in PLWH, the pathological finding associated with higher cortical expression of EphB2 was the presence of multinucleated giant cells or microglial nodules, highlighting microglia as potential key perpetrators of brain injury and neurocognitive impairment.

On the other hand, we observed that the type I interferon IFNβ, which overall exerts anti-viral and neuroprotective effects [9, 29, 30], can induce ephrin-B1/EphB2**.** Interestingly, EphB2 mediated ephrin-B1 reverse signaling on microglia in turn generates an anti-viral, type I interferon response. GO enrichment analysis of our RNA-seq data for EphB2 treated microglia vs Ctrl found some of the most enriched biological processes to include “Response to Interferon Beta”, “Regulation of Viral Life Cycle” and other cytokine responses. Differential gene expression analysis and network analysis clearly shows a broad and robust type I interferon response, including activation of IRF7, IFITM1, RIG-I, MX2, IFI35 as seen by network analysis of the second highest scoring network. Hence, it seems possible that there is a previously unrecognized positive feedback loop. Beyond the previously discussed NF-kB binding sites, transcription factor binding sites on the promotor for human EFNB1 (Genecards ID: GC0XP068828) include IRF4, IRF5 and TRIM25 (a regulator of the RIG-I viral cytoplasmic detector), while the promotor of human EPHB2 (Genecards ID: GC01P022710) includes transcription factor binding sites for IRF4, STAT5, RELB. This suggests a potential route through other IRFs, such as IRF4 or IRF5 that could be the intermediaries for IFNβ to promote ephrin-B/EphB expression.

To fend off the virus, this EphB2 induced response may provide an alternative mechanism for the host to drive an anti-viral phenotype in a seemingly positive feedback loop given ephrin-B1/EphB2s own activation by IFNβ. However, what remains unclear is what impact the chronic presence of elevated EphB2 in the CNS has on inflammation and the subsequent impact on neuronal function and survival. Acute EphB2 treatment, as shown with the RNA-seq and secretome experiments in this study, activates NF-kB signaling and induces a unique cytokines/chemokines profile, which our findings show to be associated with neurotoxicity [31, 32]. Indeed, the transfer of cell-free CM from microglia stimulated with EphB2-Fc shows non-contact dependent neurotoxicity potentially stemming from the secreted cytokine/chemokine profile. This presents itself as a potentially novel mechanism for HIV associated neuronal damage that is based on the pro-inflammatory effects of an insufficient anti-viral response. The inflammatory signature of EphB2 treated microglia overlaps with a hallmark immune signature in the brain of various HIV models and PLWH including CCL2, CXCL10, IL-1B, TNFα, IL-6, MMP-9 and others [33–35]. Of particular note, MMP-9, is upregulated in the CNS of HIV infected individuals [36] and various neuroHIV models and associated with Blood Brain Barrier leakiness through reduced vascular tight junction proteins and presumably enhanced peripheral immune infiltrates and subsequently viral entrance to the CNS [37, 38]. TNFα serves a similar role to MMP-9 by opening a para-cellular route for HIV carried by macrophages through the blood brain barrier, where chemoattractive signals from activated microglia, including CCL2 and CXCL10, further perpetuate the infiltration into the CNS [39].

Neurotoxicity is a pathological phenomenon seen in neuroHIV that was also observed through transfer of cell-free CM from Ephb2 activated microglia onto neurons, which our data suggest is a consequence of the assortment of pro-inflammatory secreted factors (IL-6, CXCL10, TNFα, IL-1β etc.). However, to control for the potential carryover of remaining active EphB2 in the microglia conditioned media, an EphB2 only control (EphB2-ctrl) containing only EphB2-fc was transferred to the iGluta/iAstrocyte co-cultures showing some limited loss of surviving neurons, but not to the extent of the EphB2-CM of microglia. The presence of other ephrins on microglia, and the partial knockdown of ephrin-B1 rather than complete deletion may explain the inability of ephrin-B1 knockdown to fully preserve neuronal survival at control level**.** However, comparison of ephrin-B1 siRNA + EphB2-CM with EphB2-ctrl, or ephrin-B1 siRNA-CM, or untreated shows no statistically significant difference in neuronal cell numbers, indicating that the knockdown of microglial ephrin-B1 may be sufficient to abrogate neurotoxicity resulting from microglial EphB2-Fc stimulation. Astrocytic ephrin-B1 was previously shown to play a role in remodeling synapses through astrocytic STAT3, potentially mediated by EphB2 signaling, therefore we cannot exclude that addition of EphB2 only to iGluta/iAstrocyte cultures caused some limited neuronal injury via astrocytes. However, microglial EphB2-CM reduced neuronal survival compared to the EphB2-control (*P* < 0.05) suggesting that secreted microglial products, likely pro-inflammatory in nature, are resulting in more pronounced, significant neurotoxicity. IL-6, for example, is elevated in microglia following EphB2 treatment but also in the brains of PLWH and is associated with HIV induced depression. Physiologically, chronic exposure of IL-6 has been shown to elicit behavioral abnormalities (seizures and ataxia) and abnormal electro-encephalogram (EEG) patterns in hippocampus [33, 40, 41]. More recent studies propose a novel mechanism by which IL-6 induces changes in neuronal iron uptake and transport pathways that result in neurodegenerative iron sequestration [42]. CXCL10 (also known as IP-10), a prominently secreted factor from EphB2 activated microglia is one of many factors we observed that have been previously described to be neurotoxic. CXCR3, the receptor for CXCL10, is present on neurons, and following CXCL10 activation in a polyinosinic-polycytidylic acid (PIC) model, CXCL10 was shown to induce hyperexcitability, with CXCR3 inhibition attenuating seizure hypersensitivity induced by PIC challenge [43, 44]. Additionally, elevated TNFα and IL-1β have been observed in the CSF of PLWH with HIV-associated dementia (HAD), two cytokines with the capacity to both induce neuronal injury via release of neurotoxic molecules, including ceramide and L-cysteine [44–46].

As with many viral infections, the immune system has to accomplish a delicate balancing act of controlling the infection without causing rampant inflammation and tissue damage. Both runaway viral activation and runaway anti-viral response need to be managed to prevent long term bystander neuronal dysfunction. Completely ablating EphB2 is likely to be a poor therapeutic approach, as the molecule serves important cellular functions in neurons, such as NMDA receptor recruitment and maintenance of healthy and mature neural spines in the hippocampus [47]. Based on the results of our knockdown experiments, targeting microglial ephrin-B1, and plausibly other microglial ephrin-B molecules, may provide a more viable option to minimize the deleterious neuroinflammation seen in the CNS in viral encephalitis stemming from HIV or potentially other neurodegenerative diseases. On the other hand, previous studies from us and others revealed that the transient expression of endogenous IFNβ limits its ability to prevent HIV/SIV-associated brain injury [9, 29]. In contrast, treatment with recombinant IFNβ completely prevented neuronal injury in the HIVgp120tg model of neuroHIV and in vitro despite the increased expression of ephrin-B1 and EphB2 [9]. This indicates that neurotoxicity associated with increased activity of the ephrin-B1/EphB2 axis can be overcome when IFNβ levels are elevated through treatment. Of note, the IFNβ treatment of the non-HIVgp120tg control mice did not cause any neuronal injury despite the upregulation of ephrin-B1 [9]. The microglial neurotoxicity induced by stimulation of the ephrin-B1/EphB2 axis in this study occurred in the absence of IFNβ, suggesting that a waning or absent IFN response may enable inflammatory neurotoxicity to ensue. Moreover, the negative feedback exerted by IRF7 on IFNβ expression may contribute to shifting the balance from neuroprotection to neuroinflammatory toxicity.

A potential limitation of the study is the use of the immortalized human HMC3 cells as the model for human microglia. While primary microglial cells have the capacity to proliferate, immortalized HMC3 may not be matching the functional spectrum of microglia under physiological conditions. The use of alternative models, including iPSC derived microglia or primary human microglia, in future studies will aim to provide improved in vitro models. Additionally, in our study, ephrin-B1 siRNA knockdown reduced expression by approximately 50%, therefore residual reverse signaling through ephrin-B1 presumably still occurs. This may explain the only partial neuroprotective effects of ephrin-B1 siRNA following EphB2 treatment when microglia CM was transferred onto iGluta/iAstrocyte co-cultures. Furthermore, EphB2 can bind to other ephrin-B and some ephrin-A variants. Therefore, it may not be surprising that simply targeting ephrin-B1 does not fully abrogate EphB2 signaling in microglia [48]. Thus, even the complete ablation of ephrin-B1 on microglia may only provide a partial signaling blockade and partial alleviation of the detrimental bystander effects on neurons stemming from EphB2-induced reverse signaling on microglia. Additionally, given the inherent variability of in vivo models, including the HIVgp120tg mouse, the limited sample size of three biological replicates per treatment in Fig. 2B may not provide sufficient statistical power to detect significant differences between groups.

In conclusion, our study highlights EphB2 as a potential biomarker to discern PLWH with and without brain pathology. EphB2 and mutual activation by ligand ephrin-B1 seem intimately linked to type I interferon signaling as shown by the robust differential expression of ephrin-B1/EphB2 on microglia following IFNβ stimulation. The apparent function of the elevated EphB2 in the CNS, at least through EphB2-mediated ephrin-B reverse signaling to microglia, is to perpetuate the anti-viral response, which includes production and secretion of pro-inflammatory products. Although the anti-viral response is crucial to reduce the viral burden, the pro-inflammatory factors are likely culprits in neurotoxicity. Microglial mediated neuroinflammation has been the suggested perpetrator for a plethora of neurodegenerative diseases besides neuroHIV, including Alzheimer’s Disease and Parkinson’s Disease [49]. This study opens the door for a novel microglial signaling pathway that could be modulated to shed light on and potentially quell the physiological, pathological, and clinical symptoms associated with these degenerative diseases that have a neuroinflammatory component.

## Materials and methods

### QRT-PCR of human samples

RNA from the middle frontal gyrus (neocortex) of HIV^+^ patients and non-infected controls were isolated and prepared by Dr. Benjamin Gelman’s laboratory (UTMB Galveston, TX) as a component of the National NeuroAIDS Tissue Consortium (NNTC). Investigators were blinded to HIV pathology status of the patients. All samples were coded, and qRT-PCR was performed as previously described [7, 9, 50]. Briefly, 500 ng of RNA from the middle frontal gyrus was reverse transcribed using SuperScript II reverse transcriptase (Invitrogen, USA, Cat# 18,064,071) following the manufacturer’s instructions. QRT-PCR was performed using Power PCR SYBR Green master mix (Applied Biosystems, Cat#: 4,367,659) on the QuantStudio 6 Flex System (Applied Biosystems, RRID:SCR_020239). The results obtained were analyzed using the 2^−ΔΔCt^ method and normalized to β-actin.

### Mouse models

The original HIVgp120tg and IFNβ-deficient mice were kindly provided by Dr. Lennart Mucke (Gladstone Institute of Neurological Disease, University of California, San Francisco, CA) and by Dr. Tomas Leanderson (Lund University, Lund, SE), respectively [8, 51]. We have recently characterized HIVgp120tg mice and crosses with IFNAR1KO and IFNβKO animals [11, 52]. Wild-type, HIVgp120tg animals and IFNβ treated animals for immunohistochemistry were all 3–4 months old and male. Intranasal treatment with recombinant mouse IFNβ was performed once a week over a 4-week time period as reported in a recent study [9]. IFNβ deficient HIVgp120tg were 9–12 months old when both males and females were analyzed [51]. All procedures involving animals were performed in accordance and compliance with the National Institute of Health *Guide for the Care and Use of Laboratory Animals* and approved by the Institutional Animal Care and Use Committees of the University of California, Riverside, and the Sanford-Burnham-Prebys Medical Discovery Institute and followed the ARRIVE guidelines.

### Immunohistochemistry of HIVgp120tg and IFNβ treated animals

Immunohistochemistry was performed according to previously published protocols from our lab [9, 52–54]. Briefly, 3–4-month-old WT, HIVgp120tg or IFNβ treated animals were terminally anesthetized and brains were quickly removed and fixed for 72 h at 4 °C in 4% paraformaldehyde (PFA) made in PBS. Brains were sectioned using a vibratome (Leica VT 1000S, Leica Biosystems, Buffalo Grove, IL, RRID:SCR_016495) to generate 40-μm-thick sagittal brain sections. Sections were subsequently stained with ephrin-B1 (R&D Systems, AF473, 1:20, RRID:AB_2293419), Iba1 (Wako, Cat#019–19741, 1:1000, RRID:AB_839504) and GFAP (Cell Signaling Technology, Cat#3670, 1:1000, RRID:AB_839504). Immunolabeled sections were mounted on glass slides with Vectashield (Vector Laboratories Inc., Newark, CA, Cat# H-1000) and overlaid with coverslips. Slides for analysis were imaged using a Zeiss LSM 880 with Airyscan Confocal Laser Scanning Microscope (Carl Zeiss AG, Oberkochen, DE, RRID:SCR_020925). Image analysis was accomplished using the ImageJ software package (ImageJ 1.53a software, RRID:SCR_003070) by taking total mean intensity of ephrin-B1 in the CA1 of the hippocampus or generating regions of interest around microglia and assessing mean intensity of ephrin-B1 of hippocampal microglia.

### Tissue culture and treatment of HMC3 microglia

HMC3 were purchased from ATCC (Cat#CRL-3304, RRID:CVCL_II76) and cultured in EMEM (Gibco), 10% FBS (Hyclone) and a combination of 100U/mL penicillin with 100ug/ml streptomycin (Sigma). HMC3 cells were cultured in 12 well (RNA) or 6 well (protein) tissue culture plates (Falcon). Cells were treated with lipofectamine RNAiMAX + ephrin-B1 siRNA (Life Technologies, ephrin-B1 silencer select Cat#4,392,420) or negative control siRNA (12 well- 12.5 pmol, 6 well- 25 pmol) for 48 h, followed by pre-clustered EphB2-Fc (R&D, Cat# 5189-B2-050, 2ug/ml) for either 24 h (RNA or secretome) or 15 min (protein lysate) before lysing the cells. Pre-clustering occurred for 1 h on ice by combining equal amounts of EphB2-Fc (R&D, Cat# 5189-B2-050) or Ctrl-Fc (R&D, Cat#110-HG) and Goat anti Human IgG (JacksonImmuno, Cat #109–005-003, RRID:AB_2337532), and mixing every 15 min. For western blot studies, cells were switched to serum free media 1 h before treatment with pre-clustered EphB2-Fc. Following EphB2 treatment for the appropriate time, supernatants were collected and stored at −80 °C for follow-up experiments, such as neurotoxicity testing. For RNA studies, cell lysis was performed on ice for 15 min with RLT buffer (Qiagen) + 1% Beta-mercaptoethanol (β-ME, Sigma). For protein studies, lysis was accomplished using a protein lysis buffer comprised of 10 mL RIPA Buffer with one tablet of protease inhibitor (Roche) and 100uL phosphatase inhibitors (Calbiochem). Treatment of HMC3 with IFNβ used human recombinant interferon beta 1a (3,000 U/ml) (PBL Assay Science, Cat#11,415–1) or 0.001% BSA (vehicle control) for 24 h, and for IRF7 knockdown studies cells were treated for 48 h with RNAiMAX + IRF7 siRNA (Life Technologies, silencer select Cat#5,194,563, 12.5 pmol) or negative control siRNA prior to IFNβ exposure.

### Isolation of mRNA and RT-PCR from mouse tissue and human cells

RNA of murine cerebral cortex was isolated using the Qiagen RNeasy Lipid Tissue Midi Kit (Qiagen) and hippocampal RNA and HMC3 RNA were isolated using Qiagen Mini Kit (Qiagen) according to the manufacturer’s instructions. QRT-PCR was performed as described above for human samples and previously reported [7, 54]. All primers are listed in Table 1. The results obtained were analyzed using the 2^−ΔΔCt^ method and relative amounts of mRNA of every gene were calculated by normalizing to the respective GAPDH/Gapdh or ACTIN/Actin house-keeping genes as internal control.

**Table 1:** qRT-PCR primers. List of forward and reverse sequences for primers used for qRT-PCR including gene target name, species, forward primer sequence, reverse primer sequence and final concentration used

Gene Name	Species	Forward	Reverse	Final Concentration
*Irf7*	Mouse	CACCCCCATCTTCGACTTCA	CCAAAACCCAGGTAGATGGTGTA	5uM
*Efnb1*	Mouse	ACCCTAAGTTCCTAAGTGGGA	CTTGTAGTACTCGTAGGGC	20uM
*Ephb2*	Mouse	TACATCCCCCATCAGGGTGG	GCCGGATGAATTTGGTCCGC	20uM
*Gapdh*	Mouse	AGGTCGGTGTGAACGGATTTG	TGTAGACCATGTAGTTGAGGTCA	20uM
*Actin*	Mouse	ACGGCCAGGTCATCACTATTG	CAAGAAGGAAGGCTGGAAAAGA	20uM
IRF7	Human	CATTCCTGGCACACACACAT	AAGCCCTTCTTGTCCCTCTC	20uM
EFNB1	Human	GTCCTACTACTGAAGCTACG	CTCTTGGACGATGTAGACAG	20uM
EPHB2	Human	GCAGTGTCCATCATGCATC	AGTACTGCAGCTCATAGTCC	20uM
IL6	Human	CTCCAGGAGCCCAGCTATGA	CCCAGGGAGAAGGCAACTG	20uM
HEAL	Human	TGCCTTTGCACAAGCTCTTC	TGCTGCAATAACCAGGTGTC	20uM
CD68	Human	TAGCTGGACTTTGGGTGAGG	CTCTCTGTAACCGTGGGTGT	20uM
IFNβ	Human	TTGACATCCCTGAGGAGATTAAGC	TTAGCCAGGAGGTTCTCAACAATAG	20uM
IFIT1	Human	GGAAACACCCACTTCTGTCTTACTG	ATTTGGATCATTTGTGCCTTGTAG	20uM
GAPDH	Human	GTCTCCTCTGACTTCAACAGCG	ACCACCCTGTTGCTGTAGCCAA	20uM
ACTIN	Human	CATGTACGTTGCTATCCAGGC	CTCCTTAATGTCACGCACGAT	20uM

### Immunoblotting

Western blotting using protein lysates from HMC3 treated cells was conducted as previously published with minor modifications [6]. Briefly, 16 µg of protein was added to 4X LDS sample buffer and 10X reducing agent (Invitrogen) and boiled for 5 min. Samples were loaded and run in a 15 well 4–12% SDS-PAGE gel (Invitrogen; Carlsbad, CA) for electrophoretic separation. Following transfer onto a PVDF membrane, the blots were blocked with 5% bovine serum albumin (BSA) solution and subsequently incubated overnight at 4˚C with primary antibodies as follows: rabbit phosphorylated NF-kB (1:1000; Cell Signaling Technology Cat# 3031, RRID:AB_330559); rabbit total NF-kB (1:1000; Cell Signaling Technology Cat# 8242, RRID:AB_10859369); mouse ephrin-B1 (1:1000; R&D Systems, Cat#AF473, RRID:AB_2293419); and GAPDH (1:20,000; Ambion Cat# AM4300, RRID:AB_437392). Membranes were then incubated with secondary antibody in 5% BSA: goat anti-rabbit (1:2000; Cell Signaling Technology Cat# 7074, RRID:AB_2099233) and goat anti-mouse (1:25,000; Pierce, Cat#1,858,413) secondary antibodies conjugated with horseradish-peroxidase. SuperSignal Dura chemiluminescent detection kit (Pierce) was used to visualize bound antibodies. Membranes were imaged using the Bio-Rad Chemidoc XRS Gel Imaging System (RRID:SCR_019690). Densitometry analysis was performed using ImageJ 1.53a software.

### Bulk RNA sequencing

RNA collected from in vitro studies with human HMC3 cells were first bioanalyzed using the Agilent 2100 Biosystem in the UC Riverside Genomics Core. Samples with RNA integrity numbers (RIN) > 7.0 were used for downstream bulk RNA sequencing at the UC San Diego (UCSD) genomics core. RNA sequencing was performed on the Illumina NovaSeq 6000, using an rRNA depletion, Paired End (PE100) and 25 M reads per sample. FASTQ files were then processed through the reference cDNA sequences for the human genome (GRCh38), which were downloaded from the Ensembl database (Release 109). Following the retrieval of this file, we utilized the Kallisto (RRID:SCR_016582) software to generate an index that facilitates rapid transcript quantification. This index was created by employing Kallisto’s index function on the downloaded cDNA FASTA file. For quantification, we executed the Kallisto quant command. The output includes estimates of transcript abundances, making it amenable to downstream analyses, including differential gene expression studies. Read counts/TPM were converted into log2Fc using the DESeq2 script including a pre-filtering step of reads < 10 [55]. Annotation was done on Galaxy (RRID:SCR_006281) using the Human Genome ChR38.109 reference. Visualization of the differentially expressed genes employed a multitude of tools/scripts including enhanced volcano script and Ingenuity Pathway Analysis (IPA) for pathway and networks analysis. P value cutoff and log2FC cutoff was set to 0.05 and 0.4, respectively. ShinyGO 0.77 was used for GO Enrichment of biological processes, using the top 300 differentially regulated genes with an FDR < 0.05 cutoff. The data discussed in this publication have been deposited in NCBI's Gene Expression Omnibus (Koury et al., 2024) and are accessible through GEO Series accession number GSE260757 (https://www.ncbi.nlm.nih.gov/geo/query/acc.cgi?acc= GSE26 0757).

### LegendPlex

Supernatants from vehicle/siRNA control, EphB2-Fc, ephrin-B1 siRNA and ephrin-B1 siRNA + EphB2-Fc treated microglia collected following 24-h incubation were used for multiplex analysis, without dilution. Three different LegendPlex panels were used, according to manufacturer’s protocol, to obtain a comprehensive inflammatory and anti-viral profile including LegendPlex Human Vascular Inflammation Panel 1 (13-plex) (BioLegend, Cat#740,551), LegendPlex Human Proinflammatory Chemokine Panel 1 (13-plex) (BioLegend, Cat#740,984) and Human Anti-Virus Response Panel (13-plex) (BioLegend, Cat#740,349). LegendPlex beads were read on a Agilent NovoCyte Quanteon Flow Cytometer Systems 4 Lasers (RRID:SCR_025831) and subsequently analyzed using BioLegend LegendPlex Data Analysis Software Suite to generate Mean Fluorescence Intensities (MFI) and calculate concentrations. Five-parameter logistic regression (5PL) was performed for each analyte assessed, and concentrations (pg/mL) were generated using standard curves.

### Neurotoxicity assay and analysis

iCell Glutamatergic Neurons (iGluta, Fujifilm CDI, Cat#1060) and iCell Astrocytes (iAstro, Fujifilm, CDI, Cat#1037) were co-cultured following the suppliers protocols at a ratio of 6:1 in black walled clear bottom 96 well plates for imaging (Corning, Cat#353,219) previously coated in 0.1% Polyethyleneimine (PEI) Solution (Sigma-Aldrich Cat# 181,978) and geltrex (Life Technologies, Cat#A1569601). The 96 wells were coated with PEI for 1 h at 37 °C, then washed and dried overnight, followed by 1 h at 37 °C of geltrex. After 7 days of culture, with 50% media changes occurring every 2–3 days, cell-free condition media from treated microglial cells, or aggregated EphB2 only control, were transferred to the co-culture for 24 h before 4% PFA fixation for 25 min at 4 °C. Cells were permeabilized with 0.2% Triton X-100 (Fisher Scientific, Cat# 50–259-99) blocked with 10% heat-inactivated goat serum and stained with Hoechst 33,342 Dye (1:150), mouse MAP-2 (Sigma-Aldrich Cat# M4403, 1:500, RRID:AB_477193) and rabbit NeuN (Millipore Cat# ABN78, 1:500, RRID:AB_10807945). Secondary Antibodies: goat anti-rabbit AF488 (Invitrogen, Cat#A11034, 1:200, RRID:AB_2576217) and goat anti-mouse rhodamine red (Jackson ImmunoResearch Labs, Cat# 115–295-146, 1:200, RRID:AB_2338766). Cells were imaged in a 96 well black walled plate with a 40X objective using Axiovert 200 M fluorescence microscope (Carl Zeiss AG, Oberkochen, DE) with a motorized stage and Slidebook software (Intelligent Imaging Innovations version 6, Denver, CO, RRID:SCR_014423). MAP-2/NeuN double positive neurons were counted and the average of the number of MAP-2/NeuN positive cells in the vehicle treatment was defined as 100% neuronal survival.

### Statistical analysis

Analysis of histopathological data, mRNA expression, Western blotting data, and correlation analysis were performed using Prism 9 software (GraphPad Software, Inc., CA, USA, RRID:SCR_002798). Comparisons of multiple groups employed either One-Way or Two-Way analysis of variance (ANOVA) followed by Tukey’s post hoc test. Comparison of two groups used student’s t-test. *P*-values < 0.05 were considered statistically significant. For human samples, Pearson correlations were calculated to determine significance of association.

## Supplementary Information

Supplementary Material 1.

## Data Availability

The RNA-sequencing data discussed in this publication have been deposited in NCBI's Gene Expression Omnibus (Koury et al., 2024) and are accessible through GEO Series accession number GSE260757 (https://www.ncbi.nlm.nih.gov/geo/query/acc.cgi). Other data generated during the study are available from the corresponding author upon reasonable request. The materials information and protocol request should be addressed to J.K. or M.K.
