# Supplementary material for: EphB2-mediated ephrin-B reverse signaling on microglia drives an anti-viral, but inflammatory and neurotoxic response associated with HIV
Source: J Neuroinflammation. 2025 Jun 30;22:171. doi: 10.1186/s12974-025-03481-9 (PMC12211399; doi:10.1186/s12974-025-03481-9)
Supplement: Supplementary file 1 — Supplementary Material 1. [file 12974_2025_3481_MOESM1_ESM.docx]

**EphB2-mediated ephrin-B reverse signaling on microglia drives an inflammatory, but anti-viral response to HIV.**

Jeffrey Koury^1^, Hina Singh^1,2^, Samantha N. Sutley-Koury^1^, Dominic Fok^1^, Xinru Qiu^1,3^, Ricky Maung^1,2^, Benjamin B. Gelman^4,5^, Iryna M. Ethell^1^, Marcus Kaul^1,2^*

^1^Division of Biomedical Sciences, School of Medicine, University of California, Riverside, 900 University Ave, Riverside, CA, 92521, USA

^2^Infectious and Inflammatory Disease Center, Sanford Burnham Prebys Medical Discovery Institute, 10901 North Torrey Pines Road, La Jolla, CA, 92037, USA

^3^Division of Genetics, Genomics and Bioinformatics, University of California, Riverside, 900 University Ave, Riverside, CA, 92521, USA

^4^Department of Pathology, University of Texas Medical Branch, 301 University Blvd, 77555-0419 Galveston, TX USA

^5^Department of Neuroscience and Cell Biology, University of Texas Medical Branch, 301 University Blvd, 77555-0419 Galveston, TX USA

***Corresponding author**: Marcus Kaul, Division of Biomedical Sciences, School of Medicine, University of California, Riverside, 900 University Ave, Riverside, CA, 92521, USA. Tel: (951) 827-7774. E-mail: [marcus.kaul@medsch.ucr.edu](mailto:marcus.kaul@medsch.ucr.edu)

**SUPPLEMENTARY FIGURES**

**Supplementary Figure 1. IRF7 knockdown potentiates IFNβ induced IRF3, long non-coding RNA HEAL and NFKB mRNA expression.** Relative RNA expression of **(A)** IRF3, **(B)** HEAL and **(C)** NFKB in HMC3 microglia following 48-hour IRF7 siRNA knockdown followed by IFNB treatment (3000U/ml). Expression normalized to GAPDH; n = 3 biological replicates per treatment. Values are Mean + SEM; FC = Fold Change; n.s. = non-significant, * P < 0.05, ** P < 0.01, *** P < 0.001, **** P < 0.0001, Two Way ANOVA followed by Tukey’s post hoc test.

**
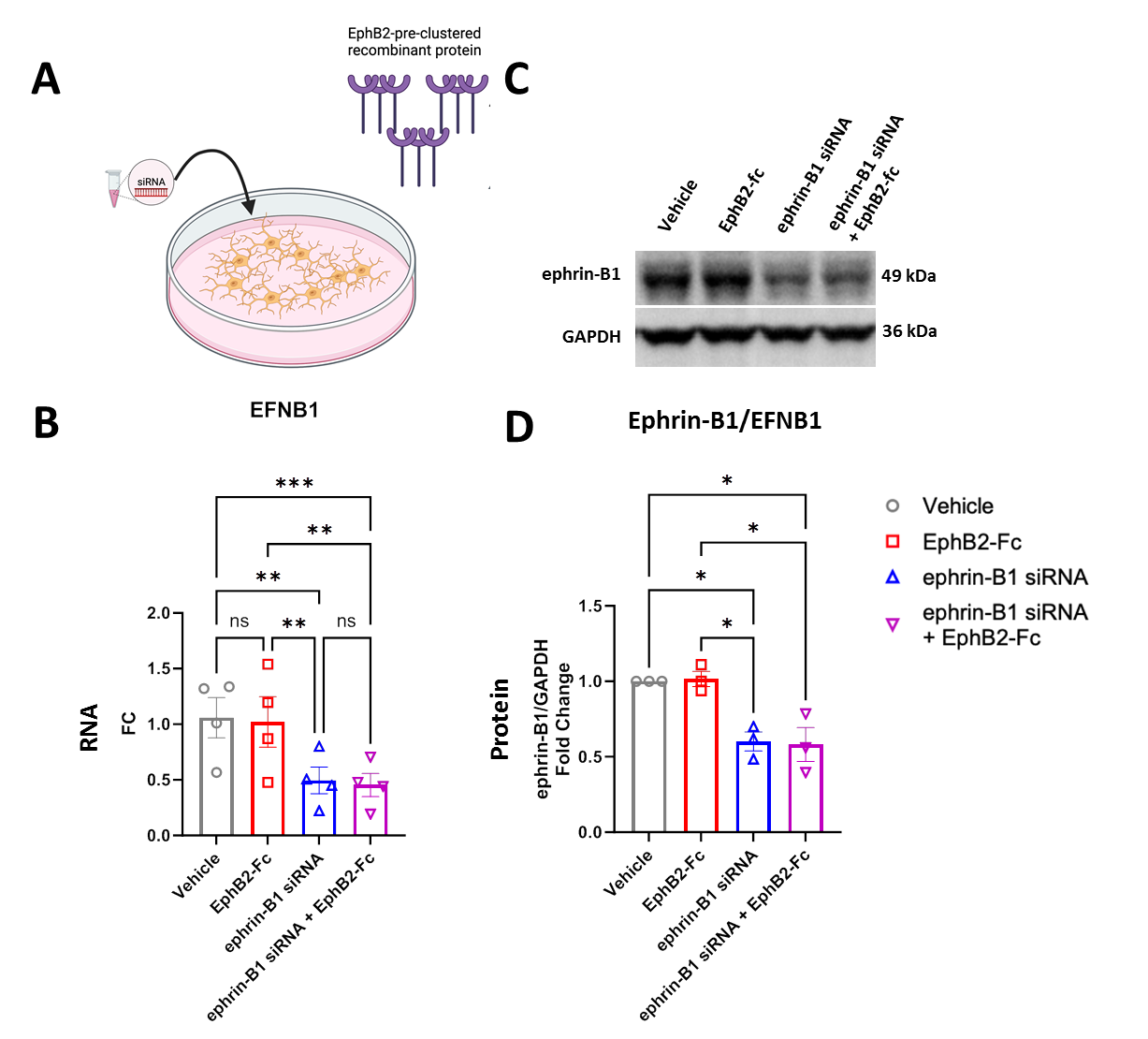
**

**Supplementary Figure 2. Ephrin-B1 siRNA downregulates ephrin-B1 at the mRNA and protein level. (A)** Schematic. **(B)** QRT-PCR analysis of EFNB1 using isolated RNA of human microglial HMC3 cells following 48-hour treatment with ephrin-B1 siRNA or scramble siRNA + 24-hour ephB2-Fc (2ug/ml) or control-Fc (2ug/ml) treatment. Fold change of EFNB1 transcript levels. **(C)** Western blot and **(D)** quantification of ephrin-B1 protein expression normalized to GAPDH; n = 3-4 biological replicates. 6-8 total technical replicates. Values are Mean + SEM; FC = Fold Change; n.s. = non-significant, * P < 0.05, ** P < 0.01, *** P < 0.001, **** P < 0.0001, Two-Way ANOVA followed by Tukey’s Post Hoc.

**
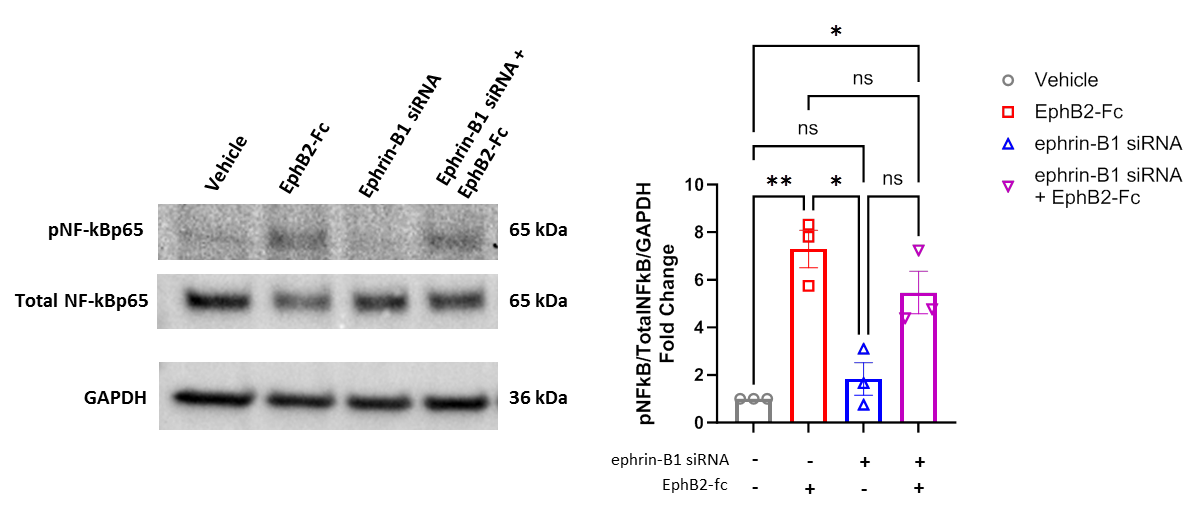
**

**Supplementary Figure 3. EphB2 treatment activates NF-kB signaling.** Representative western blot of phospho-NF-kB, total NF-kB and GAPDH. The graph shows fold change normalized to vehicle control of phospho-NF-kB normalized to Total NF-kB then to GAPDH; n = 3 biological replicates per treatment. Values are Mean + SEM with * P < 0.05, ** P < 0.01, *** P < 0.001, **** P < 0.0001, Two Way ANOVA followed by Tukey’s post hoc test.

**Supplementary Figure 4. Concentrations of secreted microglial proteins significantly differentially regulated by the ephrin-B1/EphB2 axis detected with LegendPlex assays.** Multiplexed analysis of supernatants following 48-hour treatment of ephrin-B1 siRNA or scramble siRNA + 24-hour ephB2-Fc (2ug/ml) or control-Fc (2ug/ml) treatment of human HMC3 microglia using a LegendPlex bead assay. Panels (**A-Q**) include pro-inflammatory cytokines, pro-inflammatory chemokines, and antiviral markers that were statistically significant when comparing EphB2 vs vehicle-treated cells; n = 3 biological replicates. 2 technical replicates per biological replicate averaged. Representative protein concentrations are Mean + SEM, * P < 0.05, ** P < 0.01, *** P < 0.001, **** P < 0.0001, n.s. = non-significant, Two-Way ANOVA followed by Tukey’s Post Hoc.
